# The characterization of toll‐like receptor repertoire in *Pinna nobilis* after mass mortality events suggests adaptive introgression

**DOI:** 10.1002/ece3.10383

**Published:** 2023-08-04

**Authors:** Stéphane Coupé, Ioannis A. Giantsis, Maite Vázquez Luis, Fabio Scarpa, Mathieu Foulquié, Jean‐Marc Prévot, Marco Casu, Athanasios Lattos, Basile Michaelidis, Daria Sanna, José Rafa García‐March, José Tena‐Medialdea, Nardo Vicente, Robert Bunet

**Affiliations:** ^1^ Université de Toulon, Aix Marseille Univ, CNRS, IRD, MIO Marseille France; ^2^ Faculty of Agricultural Sciences University of Western Macedonia Kozani Greece; ^3^ Instituto Español de Oceanografía (IEO, CSIC), Centro Oceanográfico de Baleares Palma de Mallorca Spain; ^4^ Department of Biomedical Sciences Fabio Scarpa, Daria Sanna: University of Sassari Sassari Italy; ^5^ Institut océanographique Paul Ricard Ile des Embiez, Var France; ^6^ Département informatique Université de Toulon Var France; ^7^ Department of Veterinary Medicine University of Sassari Sassari Italy; ^8^ IMEDMAR‐UCV, Institute of Environment and Marine Science Research Universidad Católica de Valencia SVM Calpe, Alicante Spain; ^9^ Institut Méditerranéen de Biodiversité et Ecologie marine et continentale (IMBE), Aix‐Marseille Université, CNRS, IRD, Avignon Université Avignon France

**Keywords:** adaptation, critically endangered, fan mussel, genetic diversity, *Haplosporidium pinnae*, hybrids, introgression, pathogens, toll‐like receptors

## Abstract

The fan mussel *Pinna nobilis* is currently on the brink of extinction due to a multifactorial disease mainly caused to the highly pathogenic parasite *Haplosporidium pinnae*, meaning that the selection pressure outweighs the adaptive potential of the species. Hopefully, rare individuals have been observed somehow resistant to the parasite, stretching the need to identify the traits underlying this better fitness. Among the candidate to explore at first intention are fast‐evolving immune genes, of which toll‐like receptor (TLR). In this study, we examined the genetic diversity at 14 TLR loci across *P. nobilis*, *Pinna rudis* and *P. nobilis* × *P. rudis* hybrid genomes, collected at four physically distant regions, that were found to be either resistant or sensitive to the parasite *H. pinnae*. We report a high genetic diversity, mainly observed at cell surface TLRs compared with that of endosomal TLRs. However, the endosomal TLR‐7 exhibited unexpected level of diversity and haplotype phylogeny. The lack of population structure, associated with a high genetic diversity and elevated dN/dS ratio, was interpreted as balancing selection, though both directional and purifying selection were detected. Interestingly, roughly 40% of the *P. nobilis* identified as resistant to *H. pinnae* were introgressed with *P. rudis* TLR. Specifically, they all carried a TLR‐7 of *P. rudis* origin, whereas sensitive *P. nobilis* were not introgressed, at least at TLR loci. Small contributions of TLR‐6 and TLR‐4 single‐nucleotide polymorphisms to the clustering of resistant and susceptible individuals could be detected, but their specific role in resistance remains highly speculative. This study provides new information on the diversity of TLR genes within the *P. nobilis* species after MME and additional insights into adaptation to *H. pinnae* that should contribute to the conservation of this Mediterranean endemic species.

## Graphical abstract

The endemic Mediterranean mussel *Pinna nobilis* struggles to cope with mass mortality events that are likely caused by the protozoan parasite Haplosporidium pinna. As the congeneric species *Pinna rudis* and *P. nobilis* × *P. rudis* hybris are naturally resistant to the pathogen, we performed a comparative analysis on 14 toll‐like receptor loci, between groups of resistant and susceptible individuals. Our research highlights adaptive introgression and suggests that TLR‐7 might be involved in the resistance to *H. pinnae*.

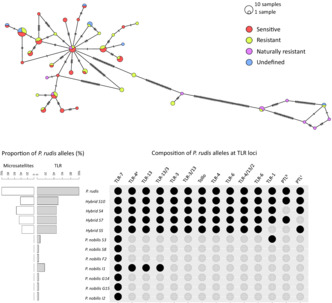

## 1 INTRODUCTION

Filtering‐feeder marine invertebrates, such as bivalves, are exposed to a wide range of potentially pathogenic microbes including bacteria, viruses, and parasites. In most cases, host and pathogens have co‐evolved over long evolutionary periods, allowing populations to cope effectively with the usual periodic epizootics (Altizer et al., 2003; Bean et al., 2013). This balance between pathogens and adapted host is currently challenged by ongoing anthropogenic changes, of which the introduction of new pathogens, global warming and pollution (Fey et al., 2015; Natalotto et al., 2015).

Newly introduced pathogens representing obvious threats for naive populations can cause dramatic demographic and genetic changes, eventually reducing biodiversity and altering ecosystem functioning and services (Elmqvist et al., 2003; Tompkins & Begon, 1999). From a biological conservation perspective, it is therefore necessary to quickly assess whether and to what extent a species is able to rapidly adapt to new infections and naturally recover (Mable, 2019). This knowledge is indeed crucial in determining the risk of global spread of pathogens, the magnitude of potential mass mortality events (MME) as well as the development of conservation strategies.

To this end, rapidly evolving immune genes are among the most relevant candidates genetic loci to study at first intention, in particular those encoding toll‐like receptors (TLR) (Grueber et al., 2015; McCallum & Dobson, 2002; Turner et al., 2011). TLR indeed have a pivotal role in the recognition of a wide diversity of pathogens, through the binding of conserved pathogen‐associated molecular patterns (PAMP) (Uematsu & Akira, 2008), as well as in the stimulation and regulation of both innate and adaptive immunity (Akira & Takeda, 2004; Barreiro et al., 2009). Previous research performed on different species has also shown that TLR genes have undergone pervasive or episodic positive selection (Fornarino et al., 2011; Grueber et al., 2015; Jiang et al., 2017; Tschirren et al., 2013), in that variations in the sequences of TLR genes could be associated with both pathogen resistance and disease tolerance (Chevillard et al., 2018; Destoumieux‐Garzón et al., 2020; Imai et al., 2008; Jiang et al., 2017; Madan‐Lala et al., 2017; Nahrendorf et al., 2021; Pila et al., 2016; Rakoff‐Nahoum et al., 2004; Tschirren et al., 2013). Moreover, determining the diversity of adaptive loci may likely help to understand the evolutionary processes underlying adaptation to pathogens and provide biomarkers that could be used to identify eventually most adapted individuals (Mable, 2019; McCallum & Dobson, 2002).

Different patterns of diversity could actually be expected according to the relative influence of selection, driven by one or multiple infectious agents for instance (Connallon & Clark, 2012; Hedrick, 1999), drift and demographic fluctuations. Selection can have antagonist effects by either maintaining or increasing diversity or reducing diversity by promoting the fixation of adaptive alleles in certain groups or local populations (Holderegger et al., 2006; Spurgin & Richardson, 2010). On the contrary, distinct scenarios of population size fluctuations can as well lead to contrasted measure of diversity (Sutton et al., 2015; Taylor & Jamieson, 2008). For instance, excess of low‐frequency variants would sign a strong long‐lasting bottleneck, while an excess of intermediate‐frequency variants would be related to a recent bottleneck of weak intensity, or to a balancing selection.

The diversity of TLRs has been mainly studied in vertebrates, globally highlighting potentially large number of haplotypes as well as high number of segregating sites (Liu et al., 2020). Noteworthy, in vertebrates, d*N*/d*S* ratios are generally less than unity, indicating that selection rather suppresses changes in protein sequences (i.e. negative or purifying selection) (Kryazhimskiy & Plotkin, 2008). Regarding mollusc taxa, less knowledge is available, but a high level of polymorphism has been evidenced overall the transcriptome of the bivalve species *Macoma balthica* (Pante et al., 2012).

The possibility of obtaining a fairly complete repertoire of full‐length coding DNA sequences is now possible thanks to next‐generation sequencing, making it possible to understand how these genes are eventually shaped by selective pressures, of which pathogens.

The Mediterranean endemic pen shell *Pinna nobilis* (Linnaeus, 1758) is an iconic species living in *Posidonia oceanica* meadows, in the coastal fringes from 0 m to 60 m depth. *Pinna nobilis* plays a central ecological role, reducing the turbidity of the water as a filtering organism, sheltering a number of symbiotic species and providing a hard substrate for the development of many epibiotic species.

In 1992, *P. nobilis* was declared a protected species with the category of vulnerable (European Council Directive 92/43/EEC), mainly to counteract intensive captures and habitat loss. These conservation measures proved efficient in several regions, for instance in France, Croatia, Spain and Italy, where populations regained vitality (Marrocco et al., 2019; Siletic & Peharda, 2003; Trigos‐Santos & Vicente, 2018). The most recent research on the dynamics and structure of the *P. nobilis* population indeed demonstrated high effective population sizes, overall genetic homogeneity between populations (i.e. based on *F*_ST_ calculations) and a potentially high level of connectivity (Nebot‐Colomer et al., 2022; Peyran et al., 2021; Wesselmann et al., 2018).

Unfortunately, the species has been experiencing mass mortality events (MME) since 2016, attributed to a multifactorial disease mainly conveyed not only to the protozoan parasite *Haplosporidium pinnae* but also to the bacteria *Vibrio mediterranei* and *Mycobacterium* sp., among potentially yet uncharacterized other pathogens, such as viruses and micro‐eukaryotes (Hamelin et al., 2019; Künili et al., 2021; Lattos et al., 2020; Scarpa et al., 2020). These MME led to dramatic demographic bottlenecks, throughout the species distribution area (Andree et al., 2020; Catanese et al., 2018; Lattos et al., 2020; Šarić et al., 2020; Vázquez‐Luis et al., 2017), that are expected to critically reduce the genetic diversity, increase population fragmentation and probably negatively influence the natural rescue effect through larval connectivity, due to reduced reproduction success (Cowen et al., 2007). Therefore, although few populations have recently been reported to be safe in some places (Cinar et al., 2021; Peyran et al., 2022), the vast majority of populations have been affected by those pathogens. As a consequence, the species has been included in the IUCN Red List as Critically Endangered (Kersting et al., 2019), and concerns have internationally resurfaced about the abilities of *P. nobilis* to cope with those new pathogenic threats and conservation actions are being set up.

Noteworthy, few observations of *P. nobilis* individuals surviving MMEs, primarily caused by *H. pinnae*, have been reported, and the phylogenetically related *Pinna rudis*, as well as the *P. nobilis* × *P. rudis* hybrids, seem to be naturally resistant to the parasite (Vázquez‐Luis et al., 2021), suggesting that their innate immunity could be efficient enough to prevent from infection development and spreading. Moreover, there is growing evidence that invertebrates, of which bivalves, are able to develop some kind of innate immune memory, that might be transmitted across generations (Arala‐Chaves & Sequeira, 2000; Green & Speck, 2018; Melillo et al., 2018; Wang et al., 2020) which would be a glimmer of hope for the conservation of the species.

In this study, our main objective was to bring new insights on the molecular mechanisms involved in the adaptation to *H. pinnae* epizootic (Salis et al., 2022). We chose a candidate approach focussing on TLR genes to test the hypothesis that TLRs are involved in such adaptation. Towards this aim, we obtained individuals (*P. nobilis*, *P. rudis* and their hybrids) from four distant geographical regions covering the largest part of all three taxa geographic ranges, sampled after epizootic diseases, in which *H. pinnae* was mainly involved. Therefore, we here described the genetic polymorphism and diversity at 14 predicted TLRs identified in the shotgun genome of *P. nobilis* (Bunet et al., 2021), which displayed classical extracellular leucine‐rich repeats (LRRs) and intracellular Toll/Interleukin‐1 receptor (TIR) domains (Gay & Gangloff, 2007). Then, we assessed whether and to what extent the observed genetic polymorphism could be associated with better MME survival.

## 2 MATERIALS AND METHODS

### 2.1 *Pinna* spp. samples

No experimentation involving removal, dislocation or killing of *Pinna* spp. individuals was performed. A nonlethal sampling method was specifically developed to avoid the manipulation of animals. A small piece (14 mm^2^, ~20 mg) of the mantle tissue was quickly excised using biopsy forceps before the fan mussel closes its valves. The piece of tissue was transferred in a tube filled with 95% ethanol and stored at −80°C until use.

Overall DNA or tissue samples of 43 adult individuals were obtained from Greece, Italy, Spain and France (Table 1, Table S1). Among those, 38 were identified as *Pinna nobilis*, 1 as *Pinna rudis* and 4 as *P. nobilis* × *P. rudis* hybrids. These individuals were sampled during or after epizootic disease mainly due to *Haplosporidium pinnae*. Dead or moribund individuals at the time of sampling are termed ‘sensitive’ (S). For convenience, the others that were alive without obvious symptom of infection are termed ‘resistant’ (R) (see Section 4). The *Pinna rudis* and hybrids individuals did not present any signs of disease and are termed ‘naturally resistant’ (NR). Dead specimen presented heavy lesions in the digestive gland connective tissue and signs of degenerative process in epithelial tissue. Moribund specimen presented half‐open valves and a retracted body (Figure S1).

**TABLE 1:** Summary of *Pinna* spp. samples.

Phenotype	Code	Species	Exposed to *H. pinnae*	Infected	Greece (*N* = 13)	Italy (*N* = 14)	Spain (*N* = 10)	France (*N* = 6)	Total
Alive, without signs of disease	R	*P. nobilis*	Yes	Yes	6	2			8
				No		3	3		6
	NR	*P. rudis*	Yes	No			1		1
		hybrids		No			4		4
Dead or with signs of disease	S	*P. nobilis*	Yes	Yes	7	8		2	17
				No		1	2^a^		3
Alive	U	*P. nobilis*	nd	No				4	4

Abbreviations: nd, not determined; NR, naturally resistant; R, resistant; S, sensitive; U, undetermined.

^a^ These individuals were sampled before the parasite reached the population, explaining why no parasite DNA could be detected (for further details see Nebot‐Colomer et al., 2022). However, these individuals died after the *H. pinnae* epizootic.

The presence of several pathogens has been investigated as described below, and using a diagnostic quantitative PCR as well for the detection of *H. pinnae*, as previously described (López‐Sanmartín et al., 2019) (Table 1). *Pinna nobilis* and *Pinna rudis* species status was all checked using mitogenome sequences (*data not shown*) (Catanese et al., 2022). Hybrid status, as well as potentially low introgressed individuals, was assessed by quantifying the proportion of *P. rudis* alleles, at toll‐like receptors and microsatellites, using neighbour‐joining phylogenies and visual inspection of multiple sequence alignments.

### 2.2 Illumina sequencing and reads filtering

Genomic DNAs were purified using the DNeasy Blood and Tissue Kit (Qiagen), following the manufacturer's instructions. Concentrations were determined by measuring the absorbance at 260 nm using Quantus (Promega) with quantifluor reactant. DNA integrity was assessed on an agarose gel at 1%.

DNAs were sequenced by Genewiz using Illumina sequencing generating paired‐end (PE) reads (2× 150 bp). The PE reads were processed as follows: PE reads were first filtered using Trimmomatic v0.36 in order to remove adapters and low‐quality leading and trailing bases (parameters: PE ILLUMINACLIP:TrueSeq‐PE.fa:2:30:10:4 LEADING:3 TRAILING:3). Mate‐pairs were then filtered using Fastp which resulted in PE reads flagged as MP and UNMERGED, which were concatenated (parameters: ‐t 4 ‐f 4 ‐T 4 ‐F 4 ‐c ‐g ‐x ‐m ‐‐include_unmerged). Finally, mate‐paired reads were filtered again using Trimmomatic v0.36 as described above including a filtering if the average base quality dropped below 20. Finally, reads of <60 nucleotides in length were discarded (parameters: PE ILLUMINACLIP:TrueSeq‐PE.fa:2:30:10:4 LEADING:3 TRAILING:3 SLIDINGWINDOW:4:15 AVGQUAL:20 MINLEN:60). FastQC was run at each step.

### 2.3 Read mapping and phasing

High‐quality paired reads (*Q* > 40) with a read length higher than 150 nucleotides were aligned against the reference shotgun genome, as well as the partial sequences of *Haplosporidium pinnae* coding for the small subunit ribosomal RNA (LC338065), *Mycobacterium* sp. coding for hsp65 (AY379077) and ITS (MN637877), *Vibrio* sp. coding for ATPaseA (MN201557), *Perkinsus mediterraneus*, *P. atlanticus* and *P. marinus* coding for the small subunit ribosomal RNA (AY486139, AF509333 and AF126013, respectively), using bwa‐mem (Li & Durbin, 2009). Sam files were processed using samtools (Li et al., 2009) in order to obtain bam files of aligned reads. The observed sequencing depth at coding DNA sequence was at least 10×. Reads that aligned the sequences of 19 neutral microsatellites, 14 TLRs and the cytoplasmic dynein (i.e. used as a nonimmune control gene), were phased using the ‘phase’ module of samtools with default parameters, and haplotypes were then inferred using SPADES (parameters: ‐k 21,33,55,77,99,127 ‐‐careful ‐‐only‐assembler) (Bankevich et al., 2012). All reconstructed sequences were visually checked using IGV (Thorvaldsdóttir et al., 2013). SNP present at less than 20% of the reads was not considered. Finally, each TLR haplotype was phased a second time using the phase module of DNAsp v6.0 allowing events of recombination.

### 2.4 TLR structure, phylogenetic analyses and ligand‐binding sites analyses

Once the haplotypes of predicted TLR gene‐coding sequences were obtained, the coding DNA sequences and predicted protein sequences were deduced using Augustus (Stanke & Morgenstern, 2005) using default parameters. Then, the protein structures were cross‐checked using SMART (Letunic et al., 2021), PROSITE, TMHMM‐2.0 (Krogh et al., 2001) and BLASTP in order to provide information about the presence of leucine‐rich repeat motifs (LRR) and Toll/IL‐1 resistance (TIR) domains which are functionally important and transmembrane domains.

Then, sequence consensus for each TLR protein was obtained from multisequence alignment using EMBOSS Cons available online (https://www.ebi.ac.uk/Tools/msa/emboss_cons/), for both *P. nobilis* and *P. rudis*. Consensus sequences were used to model the 3D structure, using I‐TASSER (Yang et al., 2015) and PyMOL (http://www.pymol.org/pymol), and to draw a phylogenetic tree depicting the relationships between TLR proteins based on multiple sequence alignments, using MEGA 11 and visualized using iTOLv6 (Letunic & Bork, 2021).

### 2.5 Genetic diversity and structure

Neutral microsatellites and TLR haplotypes were aligned with MEGA11, using CLUSTAL and MUSCLE (codon), respectively. Singletons of TLR sequences were removed. These alignments were used in DNAsp (Rozas et al., 2017) to determine haplotype diversity, which were translated into genotype data. Regarding TLR haplotypes specifically, the following diversity statistics were assessed using DNAsp (Rozas et al., 2017): the number of polymorphic sites (*S*), the average number of nucleotide differences among all alleles (*k*), the nucleotide diversity (*π*), the number of nonsynonymous (d*N*) and synonymous (d*S*) changes. For each *Pinna nobilis* TLR, median‐joining haplotype networks were drawn using PopART (http://popart.otaga.ac.nz).

For both TLR and microsatellite loci, FSTAT v2.9.3.2 (Goudet, 2001) software was used to determine allele diversity (*N*_A_), allelic richness based on a sample of eight individuals (*A*_R_), observed and expected heterozygosity (*H*_O_ and *H*_E_, respectively) and calculate estimates of *F*_IS_, with 10,000 genotype randomizations performed among samples to test for significance. Then, significant deviation from the Hardy–Weinberg equilibrium (HWE) was tested using the randomization procedure (100,000 steps in Markov Chain and 1000,000 dememorization steps) implemented in ARLEQUIN v3.5.1.2 (Excoffier & Lischer, 2010).

Prior to the genetic structure analysis, ARLEQUIN v3.5.1.2 (Excoffier & Lischer, 2010) was also used to check that the retained loci were not in linkage disequilibrium, to assess the distribution of genetic diversity by an analysis of molecular variance (AMOVA) and to calculate the pairwise genetic differentiations (*F*_ST_) among each population. The corrected significance threshold for multiple tests was set using the Benjamini–Hochberg correction procedure (Benjamini & Hochberg, 1995). Subsequently, genetic structures were depicted using principal component analysis (PCA) using R.

### 2.6 Assessment of selection acting at TLR loci

First, the evolutionary pressures acting on each TLR locus were assessed by a McDonald and Kreitman test and by estimating the Tajima's and Fu and Li′s *D* parameters using DNAsp (Rozas et al., 2017), either globally or regionally with a window length of 80 nucleotides and a step size of 20 nucleotides. Then, candidate SNPs under diversifying (i.e. positively selected sites – PSS) or purifying (i.e. negatively selected sites—NSS) selection were detected based on an outlier‐*F*_ST_ approach using BAYESCAN and a phylogenetically controlled test using a fast unconstrained Bayesian approximation model (FUBAR), accessible online from the DataMonkey server (https://www.datamonkey.org/) (Weaver et al., 2018). The analyses with BAYESCAN were implemented with a total of 150,000 iterations, a burn‐in period of 50,000 and 20 pilot runs of 10,000 iterations each and did not yield any significant results. The analysis using FUBAR (Murrell et al., 2013) was performed once potential recombination events were assessed for each TLR locus using GARD algorithm (Kosakovsky Pond et al., 2006), also accessible from the DataMonkey server. This is a recommended preprocessing step for selection inference, so that any recombination effects could be considered in the FUBAR model. Only sites under putative selection with a posterior probability higher than 0.9 were retained for further analysis. Finally, DAPC analysis was performed using *R*, in order to depict the relative contributions of SNP retaining the three to four clusters of individuals (i.e. according to their phenotypes), and with n.pca = 6; n.da = 4.

## 3 RESULTS

### 3.1 *Pinna* spp. samples and infectious status

The 43 individuals were sampled and sorted according to their phenotype at the sampling period after a mass mortality event. *Pinna rudis* and hybrids (i.e. termed naturally resistant ‘NR’) were always alive, without signs of disease, and no trace of *H. pinnae* could be detected in the genomic data. Sixteen *P. nobilis* did not present any obvious sign of disease (i.e. termed resistant ‘R’) of which eight were infected by *H. pinnae*, while 20 *P. nobilis* were dead or presented obvious signs of disease (i.e. termed sensitive ‘S’), of which 17 were infected by *H. pinnae*. Four French *P. nobilis* could not be defined as resistant (i.e. termed undefined ‘U’) since we only have the suspicion that *H. pinnae* affected the whole population (Table 1, Table S1).

Regarding the other sought microorganisms, genomic DNA of *Vibrio mediterranei* was detected in three *P. nobilis* from Greece and four *P. nobilis* from Italy, while *Mycobacterium* sp. was detected in nine *P. nobilis* from Greece, one *P. nobilis* of Italy and two *P. nobilis* from France. Both *Vibrio* and *Mycobacterium* were detected in equal proportions within the resistant and sensitive phenotypes. *Perkinsus* sp. was not detected in any specimen.

### 3.2 Candidate TLR structure and description of genetic polymorphism

Among the 14 contigs carrying TLR gene candidates, all encoded proteins exhibited the expected typical leucine‐rich regions, transmembrane domains, one or two TIR motifs, and horseshoe 3D structures. The detected open reading frames ranged from 1659 nt to 3624 nt in length.

In both *P. nobilis* and *P. rudis* species, we found one or several members of TLR‐1, ‐2, ‐3, ‐4, ‐6, ‐7, ‐13 and toll‐like families (Table S2). The phylogenetic relationships between TLR coding sequences of both species, as well as the protein domains of each TLR, are depicted in Figure S2.

Across the 14 *P. nobilis* TLR loci, a total of 228 haplotypes were observed with a mean haplotype per loci of 16.4 ± SD 8.1 and a mean haplotype diversity (*H*_d_) of 0.771 ± SD 0.166. Overall, one or a few haplotypes predominate at each TLR locus (Figure 3, Figure S3). The density of segregating sites is rather homogenous across loci, thus between both the extra and intracellular domains of the protein, with a mean of roughly 8.2 ± SD 5.8 substitutions per 1000 nt, except for the TLR‐7 in which 27 substitutions per 1000 nt was observed (Table 2).

**TABLE 2:** Genetic polymorphism of TLR loci.

		TLR locus													
		TLR‐4	TLR‐4	TLR‐4/13/2	TLR‐3	TLR‐3/13	TLR‐13/3	TLR‐13	Tollo	TLR‐7	TLR‐6	TLR‐6	TLR‐1	Protein toll‐like	Protein toll‐like
	Contig	38,093	473	17,440	21,890	67,982	7594:g1	7594:g2	50,674	12,778	48,600	39,119	21,812	84,580	39,158
	CDS length (nt)	2485	2535	2353	2571	2091	1941	1659	3606	3624	3240	3372	2013	1941	1971
*P. nobilis* sequences	*S*	22	14	21	8	7	12	11	38	99	27	24	15	12	11
	*S*/1000 nt	8.85	5.52	8.92	3.11	3.35	6.18	6.63	10.54	27.32	8.33	7.12	7.45	6.18	5.58
	d*N*/d*S*	1.20	0.75	3.75	0.60	1.33	3.00	0.57	1.24	0.90	0.50	0.71	1.50	1.00	2.67
	*h*	20	13	14	9	8	12	10	21	37	20	22	20	12	10
	AA	15	8	13	4	5	11	5	18	27	9	10	15	7	8
	*H*_d_	0.905	0.7	0.799	0.52	0.41	0.782	0.583	0.802	0.971	0.932	0.942	0.856	0.802	0.787
	*k*	3.385	1.121	6.423	0.604	0.565	1.839	1.834	6.887	22.781	5.197	3.467	2.32	2.903	2.311
	*π*	0.00136	0.00044	0.00273	0.00023	0.00027	0.00095	0.00111	0.00191	0.00629	0.00161	0.00103	0.00115	0.0015	0.00117
All sequences	*S*	118	154	97	97	95	99	60	138	151	119	130	26	128	69
	*S*/1000 nt	47.48	60.75	41.22	37.73	45.43	51.00	36.17	38.27	41.67	36.73	38.55	12.92	65.95	35.01
	d*N*/d*S*	0.72	1.50	1.87	0.62	1.46	0.60	0.88	1.06	0.86	0.41	0.85	2.25	0.56	1.88
	*h*	26	19	16	13	12	14	11	27	39	25	25	24	14	14
	AA	20	11	15	8	9	12	6	24	32	11	12	17	8	11
	Hd	0.921	0.741	0.828	0.584	0.477	0.813	0.642	0.833	0.971	0.941	0.949	0.872	0.819	0.819
	*k*	17.799	16.687	17.319	9.187	9.984	12.695	9.134	20.613	22.781	16.833	16.119	3.09	9.957	10.532
	*π*	0.00716	0.00658	0.00736	0.00357	0.00477	0.00654	0.00552	0.00572	0.00629	0.0052	0.00478	0.00153	0.00513	0.00534

Abbreviations: AA, number of translated proteins; d*N*/d*S*, ratio of nonsynonymous over synonymous substitution; h, number of haplotypes; *H*_d_, haplotype diversity; *k*, average number of nucleotide differences between sequences; *S*, number of segregating sites; *π*, nucleotide diversity per site.

**FIGURE 1:**
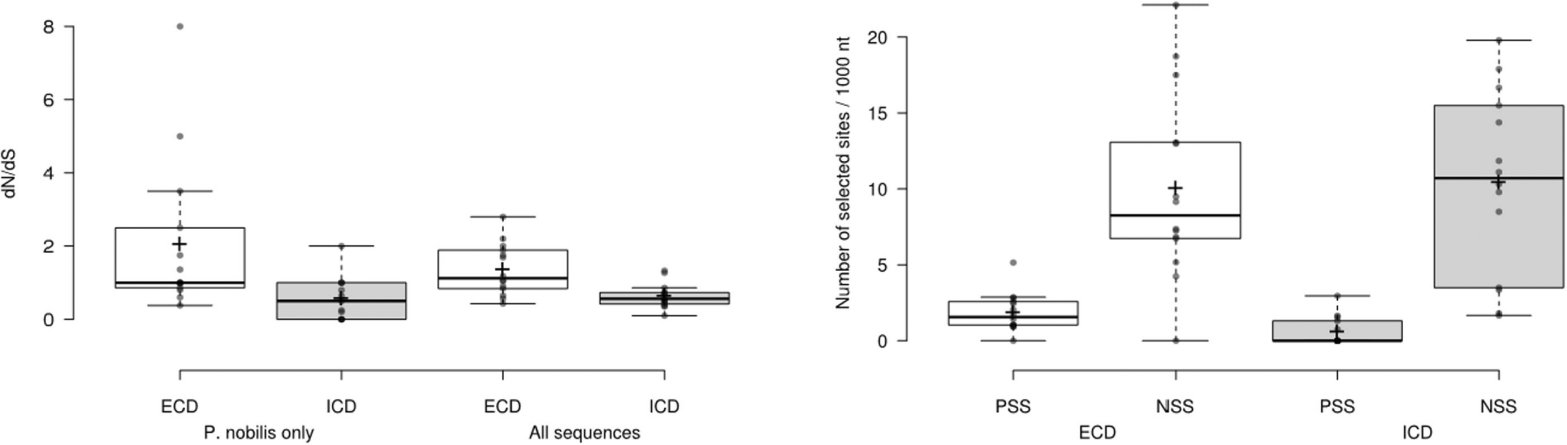
Density distribution of d*N*/d*S* ratio and positively and negatively selected sites across TLR. ECD, extracellular domain; NSS, negatively selected sites; ICD, intracellular domain; PSS, positively selected sites.

The d*N*/d*S* ratio observed at the full‐length sequences ranged between 0.5 and 3.8 (mean = 1.4 ± SD 1.0) and was mostly higher at the extracellular domain (mean = 2.1 ± SD 2.1) compared with that of the intracellular domain (mean = 0.6 ± SD 0.6), which reveals a greater polymorphism in the protein region harbouring binding sites. The observed mean number of translated proteins was 11 ± SD 6.2 (Table 2 and Figure 1).

The polymorphism observed at TLR loci was found like that observed for the nonimmune comparative control gene (i.e. 15 haplotypes, *H*_d_ = 0.714 and S/1000 nt = 9.4). However, for dynein, the d*N*/d*S* ratio was 0.2, that is three and seven times lower than the d*N*/d*S* ratios observed at intracellular domain and at the full‐length sequence of TLR, respectively.

As expected for TLR genes, the genetic polymorphism observed between orthologue sequences was rather high, with a mean density of segregating sites of 42.1 ± SD 12.6 sites per 1000 nt (Table 2).

### 3.3 Basic genetic diversity and distribution

Similar levels of genetic and gene diversities have been detected among populations as well as among phenotype groups, for both microsatellites and TLR loci. Overall, no sign of departure from Hardy–Weinberg equilibrium was observed since F_IS_ were not significant, and specifically no excess of heterozygotes could be observed (Table 3, Table S3a,b).

**TABLE 3:** Basic overall descriptive statistics.

Species	Locality	Parameters	Microsatellites	TLR	Species	Phenotype	Parameters	Microsatellites	TLR
*P. nobilis*	Greece (*N* = 13)	*N*_A_	160	131	*P. nobilis*	Resistant (*N* = 15)	*N*_A_	157	150
		*R*_A_	4.8	4.8			*R*_A_	4.7	4.87
		*H*_E_	0.748	0.743			*H*_E_	0.748	0.731
		*F*_IS_	0.074	0.047			*F*_IS_	0.070	0.034
	Italy (*N* = 14)	*N*_A_	139	132		Sensitive (*N* = 21)	*N*_A_	182	176
		*R*_A_	4.5	4.8			*R*_A_	4.7	4.98
		*H*_E_	0.741	0.747			*H*_E_	0.761	0.758
		*F*_IS_	0.020	−0.085			*F*_IS_	0.025	0.013
	France (*N* = 6)	*N*_A_	115	80	*P. rudis* & hybrids	Naturally resistant (*N* = 5)	*N*_A_	98	92
		*R*_A_	4.8	4.2			*R*_A_	5.7	5.60
		*H*_E_	0.748	0.743			*H*_E_	0.706	0.78
		*F*_IS_	−0.041	0.047			*F*_IS_	0.215	0.134
	Spain (*N* = 5)	*N*_A_	91	83					
		*R*_A_	4.4	2.6					
		*H*_E_	0.693	0.678					
		*F*_IS_	0.034	−0.137					
	All (*N* = 38)	*N*_A_	228	228		All (*N* = 41)	*N*_A_	253	277
		*H*_E_	0.777	0.766			*H*_E_	0.791	0.790
		*F*_IS_	0.031 (−0.039; 0.108)	0.001 (−0.047; 0.062)			*F*_IS_	0.058 (−0.010;0.132)	0.021 (−0.047; 0.058)

Abbreviations: *N*_A_, number of alleles; *R*_A_, allelic richness based on eight individuals; *H*_E_, expected heterozygosity; *F*_IS_: inbreeding coefficient.

**FIGURE 2:**
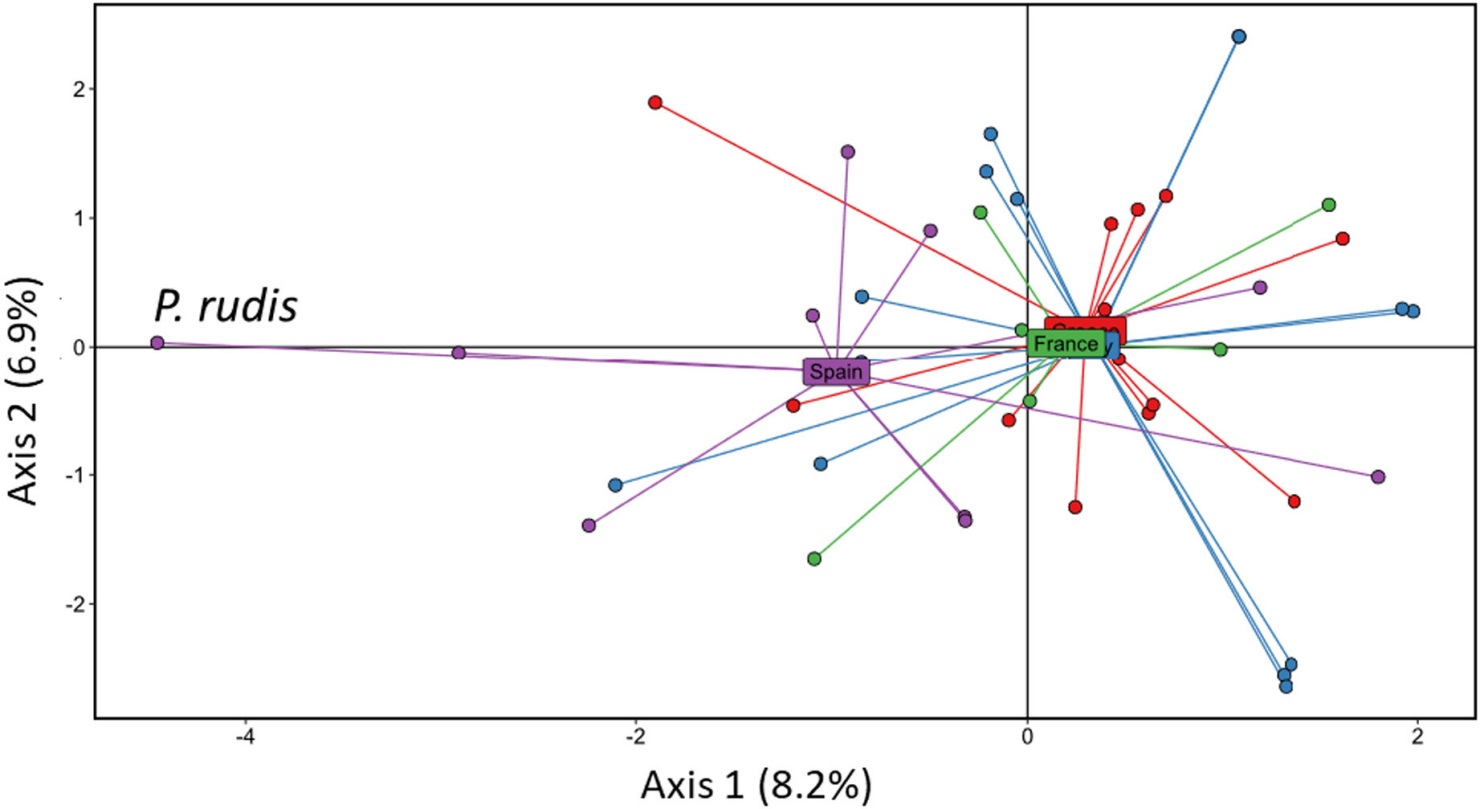
PCA performed using TLR genotypes. The analysis has been performed considering all the individuals irrespective of the species. All *Pinna nobilis* x *Pinna rudis* hybrids originated from Spain, explaining why the Spain population is remote from the three others. Individuals from Spain, Greece, Italy and France are represented in purple, red, blue and green, respectively.

The analysis of molecular variance performed considering either both species or *P. nobilis* only, all evidenced that the genetic diversities were mostly distributed within population or phenotype groups, regardless of the molecular marker considered. At the population level, the AMOVA's *F*_ST_ were significant only when both species were considered (Table S4). Consistent with the AMOVAs, pairwise‐*F*_ST_ evidenced a rather homogenous *P. nobilis* population. Using either molecular marker, none pairwise differentiation could be detected. Regarding phenotype groups, only the naturally resistant group was significantly differentiated from the resistant and sensitive groups, using both microsatellites and TLRs, which is obvious considering that the naturally resistant group is composed of *P. rudis* and hybrids individuals only (Table S5). Complementary to the *F*_ST_ analysis, PCA highlights the homogeneity of *P. nobilis* populations, and how *P. rudis* and hybrid individuals separate from *P. nobilis* individuals (Figure 2, Figure S3).

### 3.4 Assessment of selection at TLR loci

#### 3.4.1 Tests of departure from neutrality

As no genetic structure could be obviously evidenced, and to prevent from bias resulting from low population size, we considered the whole sequences as coming from one panmictic population. First, the departure from neutrality was tested using a McDonald–Kreitman test, which compares the amounts of polymorphism within *P. nobilis* and *P. rudis* species, to the divergence between them, at neutral and non‐neutral sites. Our results suggest that the TLR genes likely evolved under neutral genetic drift. At the intraspecific level, the Tajima's *D* test performed on *P. nobilis* haplotypes globally also meets the assumption of neutrality (Table 4), although there are signs (*p* < .1) of positive selection acting on TLR‐7, TLR‐4 (i.e. Contig 473) and TLR‐3. The Fu and Li′s *D* values were significantly positive for most of the TLR, evidencing overrepresented haplotype variants in the population, that may have been selected in the past (Table 4).

**TABLE 4:** Tests of departure from neutrality.

TLR	Contig	McDonald and Kreitman	Tajima's *D*	Fu and Li's *D*	d*N*/d*S* (ECD)	d*N*/d*S* (ICD)
TLR‐4	38,093	1.878	−0.73238	1.76889**	1.00	nd
TLR‐4	473	0.776	−1.70200^$^	1.55038*	0.86	0.00
TLR‐4/13/2	17,440	2.512	1.55599	1.76997**	5.00	0.00
TLR‐3	21,890	0.721	−1.57272^$^	1.27359	0.60	0.00
TLR‐3/13	67,982	1.275	−1.45349	1.20882	1.00	2.00
TLR‐13/3	7594:g1	0.81	−0.66732	1.47375^$^	8.00	0.50
TLR‐13/3	7594:g2	0.595	−0.47643	1.43053^$^	0.80	0.00
Tollo	50,674	1.21	−0.33431	2.00864**	1.36	1.00
TLR‐7	12,778	0.701	−1.58454^$^	2.08624**	0.98	0.64
TLR‐6	48,600	1.333	−0.14388	1.86558**	0.38	0.80
TLR‐6	39,119	0.839	−0.86734	1.81176**	1.00	0.20
TLR‐1	21,812	0.5	−0.66055	1.58483*	1.75	1.00
Protein toll‐like	84,580	0.99	0.55802	1.47296^$^	2.50	0.25
Protein toll‐like	39,158	2.258	0.10234	1.43053^$^	3.50	1.00

*Note*: McDonald and Kreitman test has been performed considering *P. nobilis* versus *P. rudis* alleles. Tajima's *D* and Fu and Li′s *D* have been performed considering *P. nobilis* alleles. *p‐value < .05; **p‐value < .01; ^$^.1 < *p*‐value < .05.

Abbreviations: ECD, extracellular domain; ICD, intracellular domain.

#### 3.4.2 Pattern of nonsynonymous versus synonymous substitutions

Considering full‐length sequences, 7 TLR presented a d*N*/d*S* ratio higher than 1, of which three comprised between 1.5 and 3. Figure 1 highlights that contrasted pattern of selection are likely to occur at both extra and intracellular domains. Indeed, d*N*/d*S* ratios higher than 1 are mostly encountered at the extracellular domains, suggesting that positive selection is predominant over this region of the protein and that amino acid substitutions were favoured. On the contrary, with one exception, d*N*/d*S* ratios are either equal or lower than 1 at the intracellular domains, suggesting that synonymous substitutions are favoured (i.e. neutral or purifying selection).

#### 3.4.3 Median‐joining networks of haplotypes

Overall, we observed clear star‐like phylogenies, with few high‐frequency nodes from which derive eventually rare haplotypes, which could be consistent with a population expansion model and/or a recent positive selection (Figure S3). This pattern of population expansion may reflect the increase of individuals, which is not necessarily accompanied by recovery in the loss of genetic variation.

Moreover, the networks depicted distinct phylogenies, evidencing that some TLR loci are likely under different evolutionary constraints. In line with the high level of divergence between *P. nobilis* and *P. rudis* TLR loci, the networks demonstrate a clear differentiation of naturally resistant haplotypes, separated by numerous mutational steps from sensitive, resistant or undefined ones. On the contrary, resistant and sensitive *P. nobilis* were evenly distributed across the nodes and branches, highlighting that there was no specific lineage associated with a particular phenotype, hence, no network evidenced strong purifying selection (Figure S3).

**FIGURE 3:**
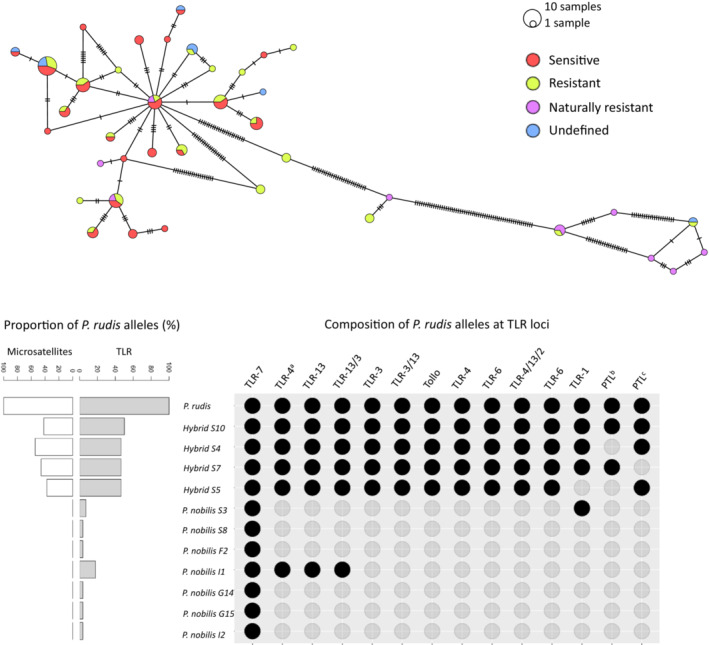
Median‐joining networks of TLR‐7 coding DNA sequence haplotypes and composition of *P. rudis* alleles at microsatellites and TLR loci within introgressed individuals. Above: Eighty‐six sequences are considered: 38, 30 and 8 haplotypes of sensitive, resistant and undefined *P. nobilis* phenotypes, respectively, and 10 haplotypes of either *P. rudis* or hybrids, naturally resistant phenotypes. The number of mutations between haplotypes is mentioned by dashes on connecting lines. Size of pie charts reflects the number of individuals with the observed haplotype. Below: Twelve and 14 loci were considered for microsatellites and TLR loci, respectively. Left: Proportion of *P. rudis* alleles within individuals. Right: Composition of *P. rudis* alleles at TLR loci. PTL, protein toll‐like, (a) contig 38,093, (b) contig 84,580, (c) contig 39,158.

However, the networks obtained for TLR‐7, ‐4, ‐13, ‐13/3 and ‐1 highlighted that 7 *P. nobilis* were introgressed with *P. rudis*, which represents about 18% of the *P. nobilis* studied here. These individuals were identified as resistant, and all carried at least a TLR‐7 allele of *P. rudis*. Strikingly, *P. nobilis* S3, S8 and F2 carried a full‐length allele of *P. rudis* whereas *P. nobilis* I1, I2, G14 and G15 carried a partial, recombined, allele of *P. rudis* (Figure 3, Figure S4). Although introgressed with *P. rudis*, G14, G15, I1 and I2 individuals were nevertheless infected with the parasite, while S3, S8 and F2 were not infected.

#### 3.4.4 Phylogeny‐based test of selection and discriminant analysis of principal component (DAPC)

Before the analysis using FUBAR, we assessed the putative recombination sites within TLR sequences, using GARD. A total of eight recombination sites were detected across six TLR sequences, mostly consisting in one recombination site detected in four TLR sequences and two recombination sites in TLR‐7 and a protein toll‐like (*data not shown*).

Among the 1481 polymorph sites observed across TLR loci (considering both *P. nobilis* and *P. rudis*), FUBAR identified 395 sites (26.6%) putatively under selection, with a posterior probability of .9. Most of these (95%) were negatively selected sites, and most of the positively selected sites (47 out of 53 sites) were found at the extracellular domain of the proteins (Figure 1).

**FIGURE 4:**
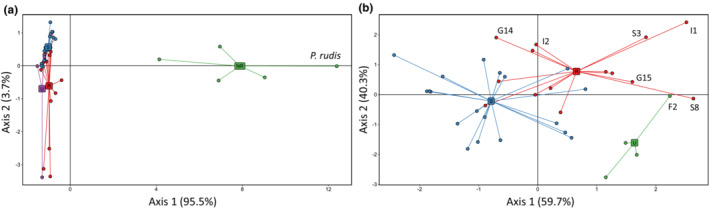
Discriminant analysis of principal component (DAPC) of the snp genotype data observed at TLR coding sequences. (a) DAPC performed according to the 1481 SNPs observed in *Pinna* spp. Naturally resistant (NR), resistant (R), sensitive (S) and undefined (U) phenotypes are represented in green, red, blue and purple, respectively. (b) DAPC performed according to the 395 SNPs detected by FUBAR as being under diversifying or pervasive selection in *P. nobilis* only. Resistant, sensitive and undefined phenotypes are represented in red, blue and green, respectively.

Complementary to FUBAR analysis, DAPC performed using either all or the putatively selected SNPs evidenced that the group of naturally resistant phenotype was obviously separated from the two others, with a probability of assignment of individuals to this cluster of 100% (*data not shown*). On the contrary, the groups of resistant and sensitive phenotypes overlapped, though few resistant individuals were remotely distributed from the others (Figure 4).

When the entire set of SNPs was used in the DAPC, almost all SNP of TLR‐4 (i.e. Contig 17,440), one SNP of TLR‐6 (Contig 48,600, snp771) and one SNP of TLR‐1 (Contig 21,812, snp1030) mainly contributed to the cluster according to axis 1, which separate the group of naturally resistant individuals from other groups. The distribution of individuals according to axis 2, which separated *P. nobilis* according to their phenotype, was driven by all SNP of TLR‐7 (i.e. Contig 12,778), eight SNPs of TLR‐4 (i.e. five SNPs for contig 17,440 and three SNPs for contig 38,093) and the snp1030 of TLR‐1, presented above.

Afterwards, the selected SNPs were used to maximize the clustering of *P. nobilis* groups only. Both axes explained a large part of the genetic variance, and the clustering was mainly driven by 13 SNPs belonging to the two TLR‐4 and the two TLR‐6. Among these SNP, only two were positively selected (TLR‐4, contig 473 snp96, and TLR4, contig 38,093 snp1108). Noteworthy, whatever the SNP contributing to the clustering, their overall contributions were weak, and no SNP could clearly segregate the resistant and sensitive groups.

### 3.5 Comparison of TLR‐7 predicted 3D structures between *P. nobilis* and *P. rudis*

**FIGURE 5:**
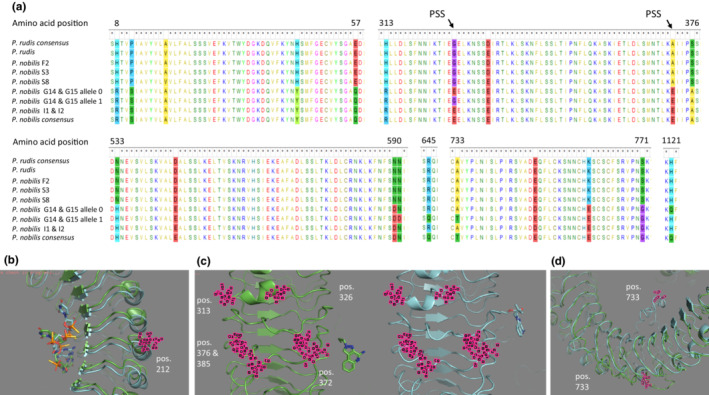
TLR‐7 polymorphism and 3D structure. (a) Sequence alignments of several regions of TLR‐7 of *P. rudis* origin within introgressed *P. nobilis*. Consensus sequences of *P. nobilis* and *P. rudis* have been included for comparison. PSS, amino acids under positive selection as detected by FUBAR. (b–d) the 3D models have been obtained using I‐TASSER software from *P. nobilis* and *P. rudis* TLR‐7 consensus sequences, represented in green and blue, respectively. (b) superimposed TLR‐7 protein at site 1 with 06S ligand; (c) *P. nobilis* and *P. rudis* TLR‐7 at site 2 with XG1 and RX8 best‐fit ligands, respectively; (d) superimposed TLR‐7 protein around site 733. Divergent amino acid substitutions are represented in pink.

I‐TASSER was used for predicting the 3D model structure of *P. nobilis* and *P. rudis* consensus TLR‐7 proteins. The software evidenced, overall, similarly shaped proteins and few 3D changes, such as the structure around the amino acids at position 733 (Figure 5). Two binding sites for guanosine analogue ligands were identified. The first one, roughly situated between amino acids 167 and 268, binds O6S ligand (PubChem reference 27,791,261). The second binding site of *P. rudis* TLR‐7, situated between amino acids 319 and 393, interacts with RX8 ligand (PubChem 159,603), while that of *P. nobilis*, situated between amino acids 369 and 443, binds the XG1 ligand (PubChem 66,958,698). Figure 5 highlights that, whatever the ligand, be it the same, the protein–ligand interactions appear specific, which are likely to induce different conformational changes and eventually protein activations.

The protein alignment of *P. rudis* and *P. nobilis* TLR‐7 consensus sequences reveals 30 amino acids differences of which 12 are associated with obvious physicochemical changes. Four of those are situated in or near the second binding sites of *P. rudis* and *P. nobilis* TLR‐7, respectively, and noteworthy two were also found under positive selection. Significant physicochemical changes due to amino acid substitutions could also be observed outside binding sites and even at the intracellular domain (Figure 5).

## 4 DISCUSSION

Determination of the evolutive capacity of threatened species is among the major keystone actions towards their conservation. Among the genes to be monitored at first intention are immune genes which act as sentinels against infectious agents. In the context of high *P. nobilis* mortality, our aim was to describe the current genetic polymorphism of the rapidly evolving toll‐like receptors, within *P. nobilis* and the phylogenetically related congeneric *P. rudis*. Such knowledge was lacking albeit the known association between TLR and fitness, resistance to diseases or survival.

The observation that *P. rudis* and hybrids were all naturally resistant to *H. pinnae* allowed us to set the hypothesis that their innate immune system was efficient to resist the infection, hence that putative‐resistant *P. nobilis* and *P. rudis* would share some genetic features associated with disease resistance, assuming that MMEs were mainly caused by *H. pinnae*.

In fact, individuals can be tolerant or resistant to a pathogen. While resistance is the ability of a host to limit parasite load or prevent infection, tolerance involves infection and is the ability to limit the negative influence on fitness for a given parasite load (Martins et al., 2019; McCarville & Ayres, 2018). Resistance and tolerance have contrasting impacts on the population dynamics of a pathogen in that resistance tends to reduce the prevalence in a population while tolerance has no influence or increases it (Kutzer & Armitage, 2016). Given interindividual variations in susceptibility to pathogens, it is conceivable that tolerance and resistance could be observed at the same time in a population.

Here, the *P. nobilis* that were interpreted as resistant individuals exhibited a longer postinfection survival time compared with those interpreted as sensitive (i.e. from few months for individuals from Greece, up to a year for those of Italy), but finally died, which may be attributed to other pathogens or factors as well. During MMEs, no regular monitoring of disease progression at the individual level could be done, so it was not possible to clearly compare the level of disease at the specimen level, nor the interindividual stress response over the course of infection. Given our samples and the lack of standardized sampling protocol, such an assessment could not be made neither. As a consequence, strictly speaking, these *P. nobilis* cannot be obviously defined as either resistant or tolerant, precisely to *H. pinnae* infection, despite the eventual inability to survive. In our analysis, for convenience, these *P. nobilis* were considered resistant.

Although we cannot exclude some assignment errors in the resistant group, we assumed that field observations were nevertheless good indicators of signs of resistance. At the molecular level, we were able to demonstrate an obvious difference between the sensitive and resistant groups since introgression at the TLR‐7 locus was observed only in the group of resistant *P. nobilis*. Additionally, three individuals of *P. nobilis* carrying the original full‐length TLR‐7 of *P. rudis* appeared as naturally resistant as the hybrids and *P. rudis* (i.e. no infection detected), suggesting that TLR‐7 was indeed probably involved in disease resistance.

This result was obtained considering a rather moderate sample size from a field study. Actually, the lack of surviving populations is a critical issue throughout the species distribution, discommoding the collection of larger number sizes of individuals, a situation that has recently been discussed (Salis et al., 2022). Nonetheless, despite the small number of TLR‐7‐introgressed naturally resistant *P. nobilis* of our dataset, this result appears highly consistent with the recent research evidencing that TLR‐7 can efficiently hamper the development of intracellular protozoan parasites (Baccarella et al., 2013; Fouzder et al., 2019; Heni et al., 2020; Regli et al., 2020). Therefore, we argue that it could rapidly help to develop conservation strategies for the species survival.

To clarify the relative importance of TLR‐7 introgression in the resistance to *H. pinnae*, further laboratory‐controlled studies should be performed. On the contrary, introgression should rapidly be more investigated in future monitoring studies as well, focussing on the TLR‐7 genetic composition of *Pinna* individuals collected either from mortality events or surviving populations. This knowledge will inform about the evolutionary potential of introgressed *P. nobilis* and the contribution of these individuals to future generations. Probably, most of the individuals identified as resistant had the same null contribution to the susceptible ones (Hasik & Siepielski, 2022). Whether the introgressed specimen contributes more to the next generation than the susceptible and the other resistant individuals, in the context of MME, needs to be assessed in future monitoring studies.

### 4.1 TLR validation and subtypes identification

Except for TLR‐4 (i.e. contig 473) and TLR‐4/2/13 (i.e. contig 17,440), all coding DNA sequences of our contigs consisted in one large exon, a feature that has been reported in other molluscs (Juhász & Lawton, 2022). All TLR coding sequences likely encoded functional receptors, as we did not observe any stop codon, and since the expected LRR and TIR motifs were always detected. The protein annotation performed using protein BLAST evidenced that the TLR repertoire we studied was composed of cell surface TLRs (i.e. TLR‐1/toll‐like protein, TLR‐2, TLR‐4 and TLR‐6) and endosomal TLRs (i.e. TLR‐3, TLR‐7/Tollo and TLR‐13) and some levels of gene redundancy. This is consistent with recent evidence that immune genes are often tandemly duplicated in molluscs and specially in bivalves (Batista et al., 2019; Peng et al., 2020; Zhang et al., 2013). Such an observation was, as well, observed on one contig (i.e. contig 7594) carrying the related TLR‐13 and TLR‐13/3.

Finally, the 14 TLR loci presented here are part, yet probably nearly complete, of the TLR repertoire of the two species. Indeed, 21 contigs were annotated as TLR in the shotgun genome of which five encoding partial TLR sequences (i.e. either LRR or TIR for instance) and two that aligned a high proportion of the same reads, which would have strongly biased the analysis.

### 4.2 Variation of TLR genes

Haplotype networks and the negative values of Tajima's *D* overall indicated that the diversity, at most TLR loci, was made of few high‐frequency haplotypes and a few to high number of rare alleles. The number of haplotypes and the level of polymorphism were similar to that reported in several vertebrate and invertebrate species (Babik et al., 2015). Densities of segregating sites observed across TLR genes were similar to that of the nonimmune control gene and are consistent with previous results obtained from the transcriptome sequencing in the bivalve *Macoma balthica* (Pante et al., 2012), which would indicate that we likely did not overestimate the level of polymorphism.

Noteworthy, TLR loci were not equally polymorphic. The observed differences in polymorphism were overall consistent with expectations in that cell surface TLRs, which bind an expanded set of ligands of different nature, exhibit high allele diversity, compared with endosomal TLRs which sense rather conserved nucleic acid structures (Georgel et al., 2009). In our study, we observed a rather unexpected high number of haplotypes at the vesicular TLR‐7 which usually presents a low diversity, at least in vertebrates. This result could be consistent with a relaxed selection acting at this locus, reflecting the high diversity of intracellular pathogens that could possibly be encountered in the environment (Künili et al., 2021; Lattos et al., 2020). Another nonexclusive explanation would be that introgression brings high levels of diversity at that locus (Fraïsse et al., 2016), which could be transmitted all in one, or partially to successive generations due to intragenic recombination. Recombination would then be a driver of diversity leading to the production of a subset of haplotypes, which then might evolve differentially than the main haplotypes. Such a contribution of recombination has already been evidenced in immune genes, including TLR (Gösser et al., 2019; Oren et al., 2019; Schaschl et al., 2006). To our knowledge, the diversity of TLR‐7 has not been described in bivalves, and a high level of TLR‐7 has just been observed in chicken (Bulumulla et al., 2011; Świderská et al., 2018). Conversely, the two other types of vesicular TLRs (i.e. TLR‐13 and TLR‐3) sense more structurally conserved double‐stranded bacterial RNAs.

### 4.3 Assessment of selection towards TLR loci

No population structure of *P. nobilis* could be detected, neither using neutral microsatellites nor TLR loci, and high and similar levels of genetic diversity were also revealed for both markers, consistent with previously published research (Peyran et al., 2021; Wesselmann et al., 2018). Moreover, considering the overall haplotypes topologies, as well as the negative Tajima's *D*, our results are in agreement with the hypothesis of a panmictic population that has been experiencing a recent postbottleneck expansion (Sanna et al., 2014) and suggest that demographic processes overweigh, or hamper the detection of a small effect of selection associated with the resistance to *H. pinnae*.

The excess of nonsynonymous mutations and high heterozygosity rates detected is, as well, congruent with at least a transient balancing selection, acting at some of the TLR loci studied (Babik et al., 2015; Fijarczyk & Babik, 2015; Minias & Vinkler, 2022). In fact, observing a clear correlation between genetic traits and a specific pathogen resistance is difficult when the populations being tested likely have contrasting life histories with respect to the microbial communities whose pathogens they have met. Accordingly, pathogens are expected to maintain high genetic polymorphisms in hosts' populations, again interpreted as balancing selection (Llaurens et al., 2017; Quéméré et al., 2018). The proposed mechanism for maintaining such a high diversity is based on either the advantage of heterozygotes (i.e. overdominance), the advantage of rare alleles (Browne & Karubian, 2018; Stefan et al., 2019), fluctuating selection (Bell, 2010; Quéméré et al., 2018) or a combination thereof. In our study, the high haplotype diversity observed mostly at cell surface TLRs and at the vesicular TLR‐7 might likely be induced and maintained by these three mechanisms, most probably by the fluctuating selection (Andree et al., 2020; Künili et al., 2021; Lattos et al., 2020; Scarpa et al., 2020) since we did not find any sign of heterozygous advantage associated to the resistance to *H. pinnae*. The other TLRs, of less polymorphism, are rather under directional selection.

The clustering analysis highlighted overall small contributions from each SNP, even though putatively selected, to the cluster, indicating that whether they had a role in the resistance or tolerance to *H. pinnae*, their effects were rather weak and more likely cumulative or synergistic. However, SNP of TLR‐7, TLR‐4 and TLR‐6 contributed the most to the clusters. At first glance, these results are in line with the scenario that *Pinna nobilis* mortalities are probably a multifactorial incidence, where the combination of various pathogens with abiotic factors may be responsible for high mortalities (Hamelin et al., 2019; Künili et al., 2021; Lattos et al., 2020; Scarpa et al., 2020), whereas specific TLRs may be triggered and favoured under combinations of factors.

In this study, we also report that hybrids are indeed significantly introgressed and that more than 18% of the *P. nobilis* of the dataset originated from ancient hybrids. Noteworthy, all *P. nobilis* we found introgressed at TLR loci were identified as resistant and carried a TLR‐7 allele of *P. rudis* origin. As discussed above, TLR‐7 is increasingly recognized as a potent modulator of protozoan infection, capable of impeding parasite growth (for an extensive review see Fouzder et al., 2019). Therefore, the fact that TLR‐7 is conserved in MME‐surviving *P. nobilis* could be interpreted as an obvious a sign of adaptive introgression (Edelman & Mallet, 2021; Gouy & Excoffier, 2020; Hedrick, 2013), but once again, there is a strong indication that the full‐length TLR‐7 of *P. rudis* is required for the effective resistance to *H. pinnae*.

### 4.4 TLR pathogens detection and immune response

TLR‐7 is an endosomal sensor of single‐stranded nucleic acids, involved in the protection against viruses, intracellular parasites and some bacteria (Baccarella et al., 2013; Barbalat et al., 2011; Fouzder et al., 2019; Heni et al., 2020; Mifsud et al., 2014; Wu & Chen, 2014). Once activated, TLR‐7 homodimers activate the interferon regulatory factors (IRF) signalling pathway and trigger both autophagy and the production of type I interferon and other inflammatory cytokines (Petes et al., 2017).

Structurally, TLR‐7 possesses two binding sites for a guanosine at site 1 and a single‐stranded RNA (ssRNA) at site 2. Both binding sites are situated at either side of a Z‐loop region which is also involved in the ssRNA recognition. Recent research evidenced that the first site was essential for TLR‐7 activation, while the second one only contributes to ssRNA‐induced activation. The TLR‐7 is thus synergistically activated in response to guanosine and ssRNA (Zhang et al., 2016, 2018). This feature of TLR‐7 likely explains why the 4 *P. nobilis* introgressed with a partial TLR‐7 of *P. rudis* could not effectively resist the infection with *H. pinnae*, whereas those with a full‐length *P. rudis* TLR‐7 were not affected.

Regarding TLR‐6 and TLR‐4, whether these TLR are indeed more or less involved in some adaptation to *H. pinnae* remains highly speculative and should require specific studies. Nonetheless, these TLR, once bound to their ligands, activate MyD88 and NFkB signalling pathways, hence triggering a pro‐inflammatory response (Krishnegowda et al., 2005; Molteni et al., 2016) which may potentialize the specific TLR‐7 response.

TLR‐6 typically binds diacylated lipopeptides (Drage et al., 2009) and glycoproteins glycophosphatidylinositol (Takeuchi et al., 2001) and has been implicated in the response and potential resistance to several pathogens, from viruses (Chen et al., 2015) to bacteria (Marinho et al., 2013) and protozoan (Leoratti et al., 2008; Netea et al., 2008). Functionally, TLR‐6 dimerizes with TLR‐2, directing specific interaction with a pathogen molecule, and hence triggers essentially an IL‐6 and TNF‐α mediated response (Krishnegowda et al., 2005). Interestingly, several research highlighted the role of TLR‐6 in mitigating *Mycobacterium* infection (Marinho et al., 2013) to which *P. nobilis* is regularly exposed to. Certain adaptive changes towards the bacteria could confer a better ability to cope with the *H. pinnae* infection as well. TLR‐4 is mostly a sensor of the bacterial lipopolysaccharide (LPS) (Qureshi et al., 1999), although a wide range of bacterial, viral and protozoan ligands have also been identified (Molteni et al., 2016; Uematsu & Akira, 2008), indicating that TLR‐4 participates in the response to a wide range of pathogens.

## 5 CONCLUSION

This preliminary research supports the role of TLR‐7 in the resistance of *P. nobilis* to *H. pinnae* and the possible influence of TLR‐6 and TLR‐4, although of much smaller effect. Further field and laboratory studies are needed to complete and clarify the role of these TLRs in disease resistance and tolerance. In this aim, a systematic and extensive characterization of health and infectious status will be required. From field research, it would be necessary to sample more *P. nobilis* that survived at least one *H. pinnae*‐driven MME and assess whether they are indeed introgressed by full‐length *P. rudis* TLR‐7. Similarly, systematically determining young recruit's haplotypes at TLR‐7 locus could, as well, help predict how resilient a population would be in the face of an *H. pinnae* epizootic. At the laboratory, controlled experimental infections, using genetically well‐characterized individuals would bring the evidence that TLR‐7 is indeed involved in the adaptation to *H. pinnae* and could also allow to assess the level of associated stress response (Box et al., 2020; Lattos et al., 2023; Natalotto et al., 2015). Moreover, prophylactic strategies to prevent infection or enhance appropriate physiological responses should be tested. Current research on this topic seems promising, by challenging the innate immune system of the host with TLR‐7 agonists which could modulate the innate immune system, thus either reducing tissue inflammation, help eliminate parasites, or prevent entry of the parasite in the host (El Hajj et al., 2018; Hamie et al., 2021). However, such experiment would probably be difficult to be performed and expensive. Finally, the fact remains that to date, no parasitic DNA has been demonstrated in *P. rudis*, hybrids or the *P. nobilis* introgressed with *P. rudis* full‐length TLR‐7, evidencing the likely pivotal role of this protein. Whether other pattern recognition receptors, eventually of *P. rudis* origin, are also involved in the protection against *H. pinnae* infection remains to be determined. The ongoing molecular research will probably bring additional insights in the mechanism of resistance.

## AUTHOR CONTRIBUTIONS

**Stéphane Coupé:** Conceptualization (equal); data curation (lead); formal analysis (lead); funding acquisition (equal); investigation (lead); methodology (lead); project administration (lead); resources (equal); software (lead); supervision (lead); validation (equal); visualization (equal); writing – original draft (lead); writing – review and editing (equal). **Ioannis A. Giantsis:** Formal analysis (equal); resources (equal); validation (equal); writing – review and editing (equal). **Maite Vázquez Luis:** Formal analysis (equal); resources (equal); validation (equal); writing – review and editing (equal). **Fabio Scarpa:** Formal analysis (equal); resources (equal); validation (equal); writing – review and editing (equal). **Mathieu Foulquié:** Formal analysis (equal); resources (equal); validation (equal); writing – review and editing (equal). **Jean‐Marc Prévot:** Software (equal); writing – review and editing (equal). **Marco Casu:** Resources (equal); validation (equal); writing – review and editing (equal). **Athanasios Lattos:** Resources (equal); validation (equal); writing – review and editing (equal). **Basile Michaelidis:** Resources (equal); validation (equal); writing – review and editing (equal). **Daria Sanna:** Resources (equal); validation (equal); writing – review and editing (equal). **José Rafa García‐March:** Resources (equal); validation (equal); writing – review and editing (equal). **José Tena‐Medialdea:** Resources (equal); validation (equal); writing – review and editing (equal). **Nardo Vicente:** Resources (equal); validation (equal); writing – review and editing (equal). **Robert Bunet:** Conceptualization (equal); formal analysis (equal); funding acquisition (equal); investigation (equal); methodology (equal); project administration (equal); resources (equal); validation (equal); writing – review and editing (equal).

## FUNDING INFORMATION

This work was supported by the University of Toulon and Toulon Provence Méditerranée (TPM) related to the PINORES project, the University Institute of Technology of the University of Toulon under the grant ‘CARTT’ and by the European Union's LIFE programme through the project LIFE PINNARCA (NAT/ES/001265). Fabio Scarpa, Marco Casu and Daria Sanna acknowledge the support of NBFC to the University of Sassari, funded by the Italian Ministry of University and Research, PNRR, Missione 4, Componente 2, ‘Dalla ricerca all'impresa’, Investimento 1.4 Project CN00000033.

## CONFLICT OF INTEREST STATEMENT

The authors declare that the research was conducted in the absence of any commercial or financial relationships that could be construed as a potential conflict of interest.

## Supporting information


Appendix S1

## Data Availability

Data referring to predicted coding DNA sequences of toll‐like receptors and genotypes at microsatellite loci are accessible through the Dryad https://doi.org/10.5061/dryad.z8w9ghxgn.
