# Supplementary material for: The characterization of toll‐like receptor repertoire in Pinna nobilis after mass mortality events suggests adaptive introgression
Source: Ecol Evol. 2023 Aug 4;13(8):e10383. doi: 10.1002/ece3.10383 (PMC10401143; doi:10.1002/ece3.10383)
Supplement: Supplementary file 1 — Appendix S1 [file ECE3-13-e10383-s001.docx]

**APPENDIX : Supplementary data**

**The characterization of toll-like receptor repertoire in *Pinna nobilis* after mass mortality events suggests adaptive introgression.**

Stéphane Coupé^1^, Ioannis A. Giantsis^2^, Maite Vázquez Luis^3^, Fabio Scarpa^4^, Mathieu Foulquié^1,5^, Jean-Marc Prévot^6^, Marco Casu^7^, Athanasios Lattos^2^, Basile Michaelidis^2^, Daria Sanna^4^, José Rafa García-March^8^, José Tena-Medialdea^8^, Nardo Vicente^9^ & Robert Bunet^5^.

1. Université de Toulon, Aix Marseille Univ, CNRS, IRD, MIO, Marseille, France.

2. Faculty of Agricultural Sciences, University of Western Macedonia, Hellas.

3. Instituto Español de Oceanografía (IEO, CSIC), Centro Oceanográfico de Baleares. Muelle de poniente s/n, 07015. Palma de Mallorca, Spain.

4. Fabio Scarpa, Daria Sanna: University of Sassari, Department of Biomedical Sciences, 07100 Sassari (Italy).

5. Institut océanographique Paul Ricard, Ile des Embiez, Var, France.

6. Département informatique, Université de Toulon, Var, France.

7. University of Sassari, Department of Veterinary Medicine, 07100 Sassari (Italy).

8. IMEDMAR-UCV, Institute of Environment and Marine Science Research, Universidad Católica de Valencia SVM, Calpe, Alicante, Spain.

9. Institut Méditerranéen de Biodiversité et Ecologie marine et continentale (IMBE), Aix-Marseille Université, CNRS, IRD, Avignon Université, France.

**Suppl. Figure 1.** Moribund *Pinna nobilis* from Thau, France (© Mathieu Foulquié).

The red arrow presents half-open valves and a retracted body.

**Suppl. Figure 2.**

Neighbour-Joining phylogenetic tree of TLR consensus sequences. The protein motifs presented are those detected using Prosite. Similar patterns were deduced using Blastp and SMART. Transmembrane domains were detected in all TLR using TMHMM.

**Suppl. Figure 3. PCA performed using microsatellite genotypes.**

The analysis has been performed considering all the individuals irrespective to the species. All *Pinna nobilis* x *Pinna rudis* hybrids originated from Spain, explaining why the Spain population is remote from the three others. Individuals from Spain, Greece, Italy and France are represented in purple, red, blue and green, respectively.

**Suppl. Figure 4.**

Haplotype phylogenies of each predicted TLR coding sequences. Smallest circles correspond to one individual.

TLR-1 (i.e., contig 21812)

TLR-13/3 (i.e., contig 67982)

TLR-4/1/2/3 (i.e., contig 473)

TLR-13/3 (i.e., contig 7594g1)

TLR-13/6 (i.e., contig 7594g2)

TLR-7 (i.e., contig 12778)

TLR-2/13 (i.e., contig 17440)

TLR-3/13 (i.e., contig 21890)

TLR-4 (i.e., contig 38093)

TLR-6 (i.e., contig 39119)

Toll-like protein (i.e., contig 39158)

TLR-6 (i.e., contig 48600)

Tollo (i.e., contig 50674)

Toll-like protein (i.e., contig 84580)

**Suppl. Table 1. Sample information.**

**Suppl. Table 2. BLASTp-based annotation.**

**Suppl. Tables 3a. *Pinna nobilis* basic genetic diversity parameters (locality)**

**Suppl. Table 3b. *Pinna* spp. basic genetic diversity parameters (phenotype)**

**Suppl. Table 4. AMOVA**

**Suppl. Table 5. Genetic differentiation**

F_ST_ values and associated p-values are presented below and above the diagonal, respectively. Significant F_ST_ are presented in bold.
